# Estimating the Global Geographical Distribution Patterns of the Invasive Crop Pest *Diuraphis noxia* Kurdjumov under Current and Future Climatic Scenarios

**DOI:** 10.3390/insects14050425

**Published:** 2023-04-29

**Authors:** Kaiting Jing, Ming Li, Haoxiang Zhao, Jianyang Guo, Nianwan Yang, Ming Yang, Xiaoqing Xian, Wanxue Liu

**Affiliations:** 1 State Key Laboratory for Biology of Plant Diseases and Insect Pests, Institute of Plant Protection, Chinese Academy of Agricultural Sciences, Beijing 100193, China; 2 Western Agricultural Research Center, Chinese Academy of Agricultural Sciences, Changji 831100, China

**Keywords:** *Diuraphis noxia*, invasive crop pest, potential suitable area, MaxEnt, climate change

## Abstract

**Simple Summary:**

*Diuraphis noxia* Kurdjumov is one of the most destructive invasive crop pests, reducing crop yield and quality and posing a great threat to global food security. Estimating the global geographical distribution patterns of *D. noxia* under climatic change is critical for its early warning and control and global food security. Here, the optimized MaxEnt model was used to predict the potential global geographical distribution of *D. noxia*. Our results showed that the potential geographical distribution of *D. noxia* was mainly located in Asia, Europe, and North America, showing a general tendency to move to higher latitudes under future climate. The increased suitable area of *D. noxia* was mainly located in northwestern Asia, western Europe, and North America. The development of ecomanagement and cross-country early warning strategies is warranted in the above area to mitigate the future impacts of the expansion of *D. noxia* on food security.

**Abstract:**

Invasive crop pests (ICPs) are a major cause of crop losses and adversely affect global food security. *Diuraphis noxia* Kurdjumov is a significant ICP that feeds on the sap of crops, reducing crop yield and quality. Although estimating the geographical distribution patterns of *D. noxia* under climate change is critical for its management and global food security, such information remains unclear. Based on 533 global occurrence records and 9 bioclimatic variables, an optimized MaxEnt model was used to predict the potential global geographical distribution of *D. noxia*. The results showed that Bio1, Bio2, Bio7, and Bio12 were significant bioclimatic variables that influenced the potential geographical distribution of *D. noxia*. Under current climatic conditions, *D. noxia* was mainly distributed in west-central Asia, most of Europe, central North America, southern South America, southern and northern Africa, and southern Oceania. Under the SSP 1-2.6, SSP 2-4.5, and SSP 5-8.5 scenarios for the 2030s and 2050s, the potential suitable areas increased, and the centroid migrated to higher latitudes. The early warning of *D. noxia* in northwestern Asia, western Europe, and North America should be attended to further. Our results provide a theoretical basis for early monitoring and warning of *D. noxia* worldwide.

## 1. Introduction

In recent years, pest invasion events have become frequent, and invasive crop pests (ICPs) have been reducing crop yields and causing enormous economic losses by feeding on crops, sucking plant sap, and spreading pathogens to crops, increasingly threatening global food security and agricultural development [1,2,3,4,5]. Globally, ICPs cause economic losses of up to USD 70 billion annually, resulting in yield losses of more than 40% [6]. Invasive aphids are important ICPs having various hosts, mainly damaging wheat, sorghum, sugarcane, and other crops; additionally, they are one of the most invasive phytophagous insects, which are widely distributed across all temperature environments worldwide [7,8,9,10,11]. For example, almost every aphid population on the Hawaii Islands is an invasive species that causes devastating attacks on the Hawaiian ecosystem [12]. Invasive aphids not only directly affect crop growth and development, leading to yield losses, but also transmit viruses to crops, which can cause serious agricultural economic losses [10,13,14,15]. To make matters worse, climate change can affect the potential geographical distribution patterns of ICPs, expanding their suitable areas and further threatening crop yields [16,17,18]. Therefore, invasive aphids pose an increasingly significant threat to crops in the context of climate change.

*Diuraphis noxia* Kurdjumov (Hemiptera: Aphididae) is one of the significant invasive aphids and among the 250 most destructive aphids in the world [9,10,14,19]. It is native to the Middle East, southern Russia, and Asia Minor [20] and can migrate over short distances through wind; additionally, it can be transported over long distances through cargo transportation [21,22]. It invaded South Africa in 1978 [23], Mexico in 1980 [24], the USA in 1986 [25], and Australia in 2016 [26]. *Diuraphis noxia* is now distributed in major wheat-growing regions on six continents [27]. It is a relatively mixed feeder with hosts that include 40 genera and more than 140 crop and weed species [28]. Wheat and barley are the main crops affected by *D. noxia*, and its infestation can affect crop growth and development, causing yield losses of more than 80% in severe cases [21,29,30]. Moreover, *D. noxia* can transmit various viruses, causing serious economic losses; within seven years of its invasion into the USA, it caused losses of USD 800 million in the western USA [31,32,33,34]. Owing to its potential for damaging crops and causing economic losses [33,34], there is an urgent need to study the potential global geographical distribution of *D. noxia*. However, *D. noxia* potential geographical distribution patterns on a global scale remain unclear. Therefore, estimating the potential global geographical distribution of *D. noxia* is crucial for early warning and management in the context of climate change worldwide. 

Species distribution models (SDMs) are important tools for predicting the potential geographical distribution of species in natural environments based on their occurrence records and related environmental factors [35]. The maximum entropy (MaxEnt) model has been widely used in the risk assessment of ICPs owing to its advantages of small sample requirement, short running time, and high prediction accuracy [36,37,38,39]. For instance, the invasion risk areas of the ICPs *Linepithema humile* (Mayr, 1868) (Hymenoptera: Formicidae) and *Helicoverpa zea Helicoverpa zea* (Boddie, 1850) (Lepidoptera: Noctuidae) in China were estimated using MaxEnt [40,41]. However, the default MaxEnt parameters are prone to model overfitting [42]. The ENMeval package in R software solves this problem by optimizing the important parameters of the MaxEnt model.

In the present study, based on the global occurrence records of *D. noxia* and related bioclimatic variables, an optimized MaxEnt model was used to predict the potential global geographical distribution of *D. noxia*. We hypothesized that the potential global geographical distribution of *D. noxia* will expand and migrate to higher latitudes in the future. Therefore, we (1) analyzed the important bioclimatic variables affecting the potential global geographical distribution of *D. noxia*, (2) predicted the potential global geographical distribution of *D. noxia* under current and future climatic scenarios, and (3) elucidated potential suitable area changes and centroid migration of *D. noxia* under future climatic scenarios. The study could provide a targeted scientific basis for early monitoring and warning by predicting the trends of the potential global geographical distribution of *D. noxia*.

## 2. Materials and Methods

### 2.1. Global Occurrence Records of Diuraphis noxia

Global occurrence records of *D. noxia* were collected from the Invasive Species Compendium of the Center for Agriculture and Bioscience International (CABI, accessed on 1 August 2022), Barcode Index Number (BOLD, accessed on 1 August 2022), Global Biodiversity Information Facility (GBIF, accessed on 1 August 2022), and China National Knowledge Infrastructure (CNKI, accessed 1 August 2022).

**Figure 1:**
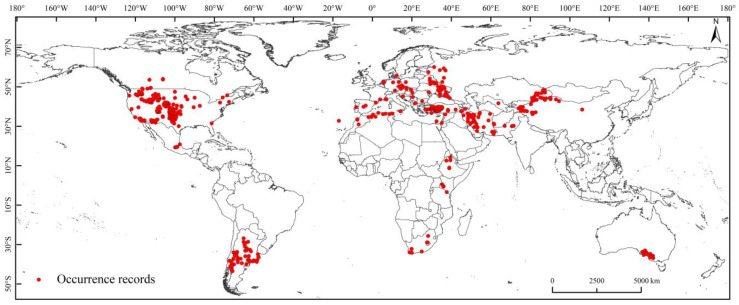
Global occurrence records of *Diuraphis noxia*.

We used ENMTools to remove redundant species occurrence records to avoid model overfitting, retaining only one occurrence record for each 5 km × 5 km raster. Finally, 533 occurrence records were retained to construct the MaxEnt model (Figure 1).

### 2.2. Selection of Bioclimatic Variables

In the current (1970–2000) and future (2030s and 2050s) climatic scenarios, data for 19 bioclimatic variables were obtained from WorldClim (https://WorldClim.org/) with a resolution of 2.5′ (Table S1). Future bioclimatic variables in the 2030s and the 2050s included three shared socioeconomic pathways (SSP1-2.6, SSP2-4.5, and SSP5-8.5) in the BCC-CSM2-MR climate system model (Figure S1). Multicollinearity among bioclimatic variables can lead to model overfitting. Therefore, we first eliminated the bioclimatic variables with zero contribution via the MaxEnt model with 10 replicate runs based on *D. noxia* global occurrence records and 19 bioclimatic variables, and we used ENMTools to analyze the correlation of the 19 bioclimatic variables. If the absolute value of the correlation coefficient between two bioclimatic variables was greater than 0.8 (|r| ≥ 0.8) [38], smaller contributing bioclimatic variables were removed. Finally, 9 bioclimatic variables were used to construct the MaxEnt model (Table 1). We used the MaxEnt model to predict the potential global geographic distribution of *D. noxia* under current and future (in the 2030s and 2050s under the SSP1-2.6, SSP2-4.5, and SSP5-8.5) climate scenarios.

### 2.3. Model Optimization, Construction, and Evaluation

The feature combination (FC) and regularization multiplier (RM) are the two most important parameters for calibrating the MaxEnt model. The feature combinations (FCs) have five features: linear (L), quadratic (Q), hinge (H), product (P), and threshold (T). The RM was set to 8 levels, from 0.5 to 4, in increments of 0.5. To avoid overfitting of the default MaxEnt model, we used the ENMeval package in the R software to optimize the RM and FCs, thereby improving model performance [42]. Finally, we selected the parameter combination of the minimum delta AICc (AICc: Akaike Information Criterion Correction, delta AICc = 0) for constructing different MaxEnt models under current and future (in the 2030s and 2050s under the SSP1-2.6, SSP2-4.5, and SSP5-8.5) climatic scenarios.

All the prediction results of the model were Cloglog, and the replicated run type was Bootstrap. Further, 25% of the species’ global occurrence records were set as the test set, and the remaining 75% of global occurrence records were used as the training set [43]. The maximum iterations was set to 500 and the max number of background points was set to 10,000 with 10 repetitions [44]. We used the area under the receiver operating curve (AUC) to evaluate the MaxEnt model performance. The AUC value ranged from 0 to 1, with a higher value indicating more accurate model prediction results [45]. In the present study, the assessment criteria of the AUC value to model accuracy were classified into three grades: AUC < 0.5, poor; 0.5 < AUC ≤ 0.8, moderate; and 0.8 < AUC ≤ 1.0, excellent [40].

### 2.4. Threshold Classification and Centroid Migration 

The results of the MaxEnt model represented the presence probability of *D. noxia* with a value range of 0–1. The ASCII format was imported into the ArcGIS software and converted into the raster format. Based on the values of maximizing sensitivity and specificity in the predicted results, the potential suitable areas of *D. noxia* were classified into four classes in the current and future climatic scenarios: unsuitable area (0 ≤ *p* ≤ 0.29), poorly suitable area (0.29 < *p* ≤ 0.40), moderately suitable area (0.40 < *p* ≤ 0.60), and highly suitable area (0.60 < *p* ≤ 1) [46].

We used the “feature to point” tool in ArcGIS software to obtain the centroids of potential suitable areas under the current and future climatic scenarios, and we analyzed the global centroid migration of the potential suitable areas of *D. noxia.*

## 3. Results

### 3.1. Model Performance

**Figure 2:**
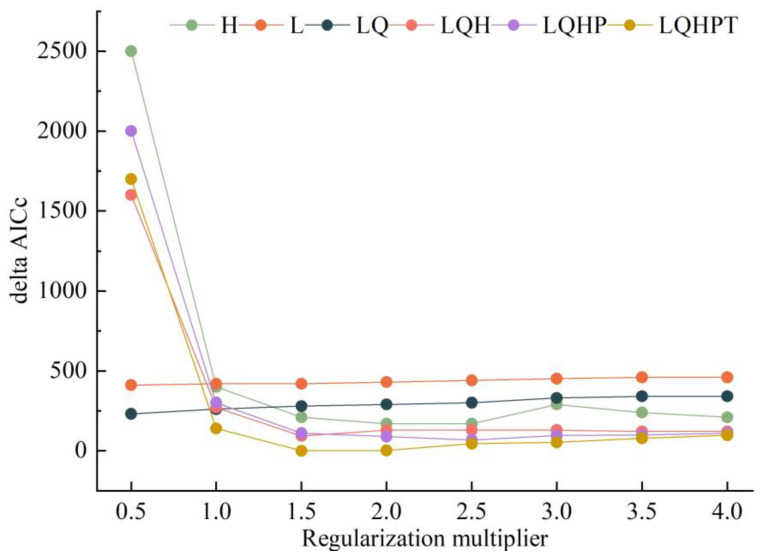
Performances of the native niche model of *Diuraphis noxia* under different settings.

**Figure 3:**
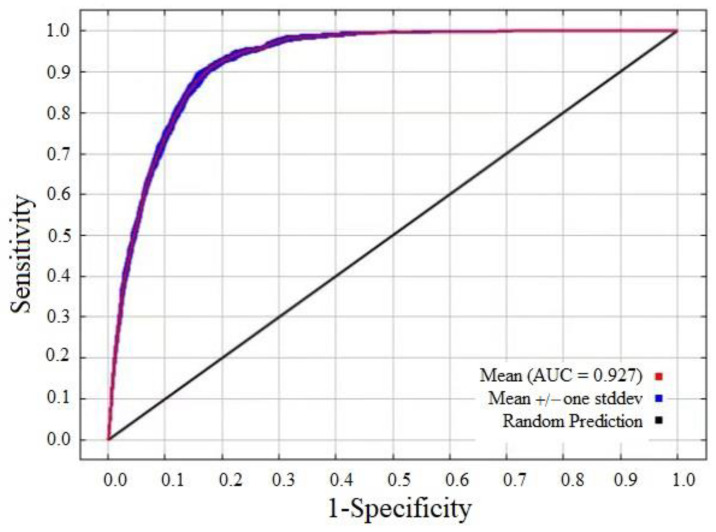
MaxEnt model receiver operating characteristic curve of *Diuraphis noxia*.

Based on the optimization results, a feature parameter combination with a delta AICc value of 0 was selected for model optimization (RM = 1.5, FC = LQHPT; Figure 2). Based on 533 global occurrence records and 9 bioclimatic variables of *D. noxia*, the optimized MaxEnt model was used to predict the potential global geographical distribution of *D. noxia.* The model prediction results showed the mean AUC value of 0.927, indicating the excellent performance of the optimized MaxEnt (Figure 3).

### 3.2. Significant Bioclimatic Variables

**Figure 4:**
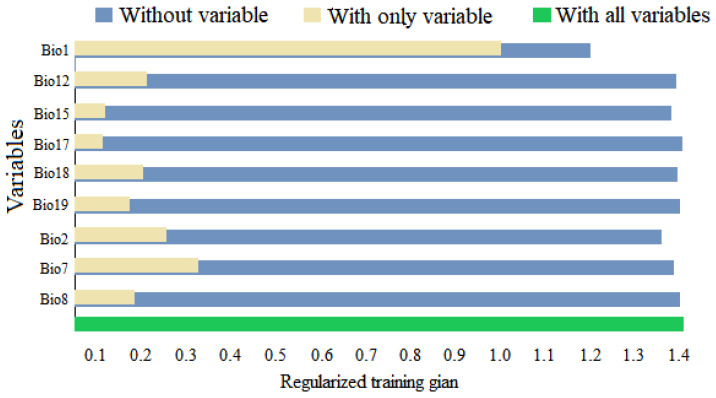
Jackknife test results of the bioclimatic variables for *Diuraphis noxia*.

We used the combination of contribution rates and the “jackknife method” to identify the degree of influence of bioclimatic factors on the distribution of *D. noxia*. Based on the results of the “jackknife method” (Figure 4), the annual mean temperature (Bio1), mean diurnal temperature range (Bio2), and temperature annual range (Bio7) were the most significant bioclimatic variables affecting the potential global geographical distribution of *D. noxia.* The bioclimatic variables with the most significant contribution rates were annual mean temperature (Bio1, 65.8%), annual precipitation (Bio12, 8.7%), and mean diurnal temperature range (Bio2, 7.1%) (Figure S2). Thus, the annual mean temperature (Bio1), mean diurnal temperature range (Bio2), temperature annual range (Bio7), and annual precipitation (Bio12) were significant bioclimatic variables for predicting the potential global geographical distribution of *D. noxia*.

**Figure 5:**
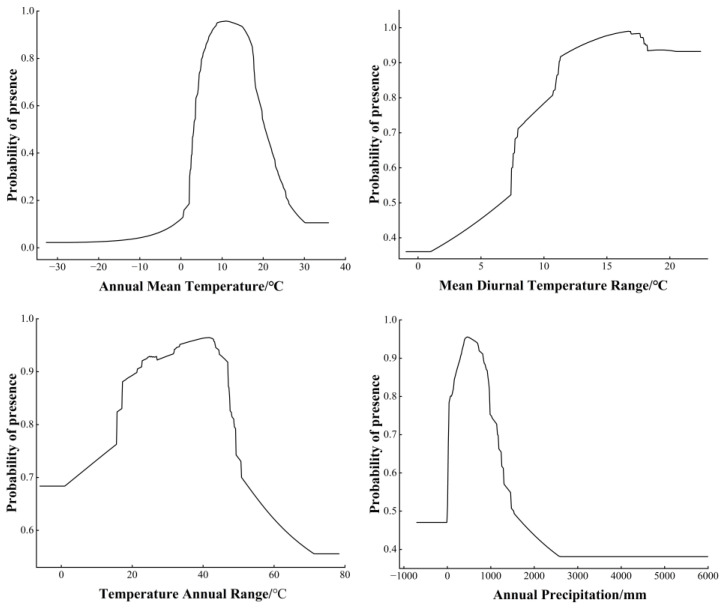
Response curves of the significant bioclimatic variables for *Diuraphis noxia*.

The response curves indicate the relationship between bioclimatic variables and the probability of species presence. When the presence probability was >0.6, the bioclimatic variables were considered more suitable for survival. Our results showed that the presence probability of *D. noxia* increased and then decreased with increasing annual mean temperature (Bio1), temperature annual range (Bio7), and annual precipitation (Bio12), whereas the mean diurnal temperature range (Bio2) increased and the probability of *D. noxia* presence showed an increasing trend (Figure 5).

### 3.3. Potential Global Geographical Distribution under the Current Climatic Scenario

**Figure 6:**
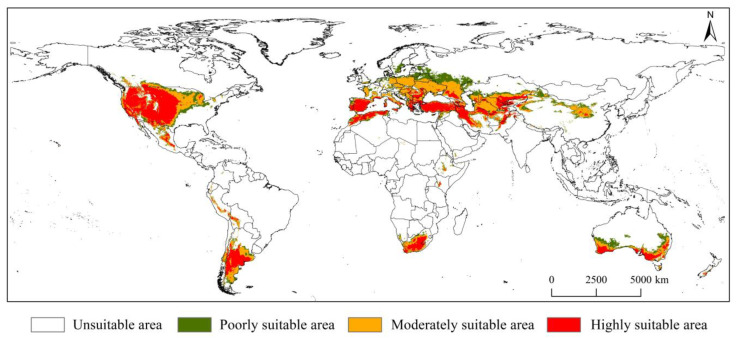
Potential suitable areas of *Diuraphis noxia* under current climatic scenarios.

The potential global geographical distribution of *D. noxia* under current climatic conditions is shown in Figure 6. The results showed that *D. noxia* was mainly distributed in central and western Asia (Kazakhstan, Uzbekistan, and Iran), most of Europe (Russia, Ukraine, and Spain), central and southwestern North America (Mexico and USA), southern South America (Argentina and Chile), southern and northern Africa (South Africa, Morocco, and Algeria), and Oceania (Australia and New Zealand). The total suitable area of *D. noxia* was 2353.05 × 10^4^ km^2^, accounting for 15.32% of the global land area (excluding the land area of Antarctica); moreover, the highly, moderately, and poorly suitable areas were 899.03 × 10^4^, 873.31 × 10^4^, and 580.70 × 10^4^ km^2^, respectively, accounting for 38.21%, 37.11%, and 24.68% of the total suitable area, respectively (Table S2). Highly suitable areas were mainly distributed in Asia (Iran, Uzbekistan, and China), Europe (Turkey and Spain), North America (the USA), and South America (Argentina). Moderately suitable areas were mainly distributed in Europe (Poland, Czech Republic, and Ukraine) and North America (the USA). Poorly suitable areas were distributed mainly in Europe (Russia, Estonia, and Lithuania), North America (the USA), and Oceania (Australia).

### 3.4. Potential Global Geographical Distribution under Future Climatic Scenarios

**Figure 7:**
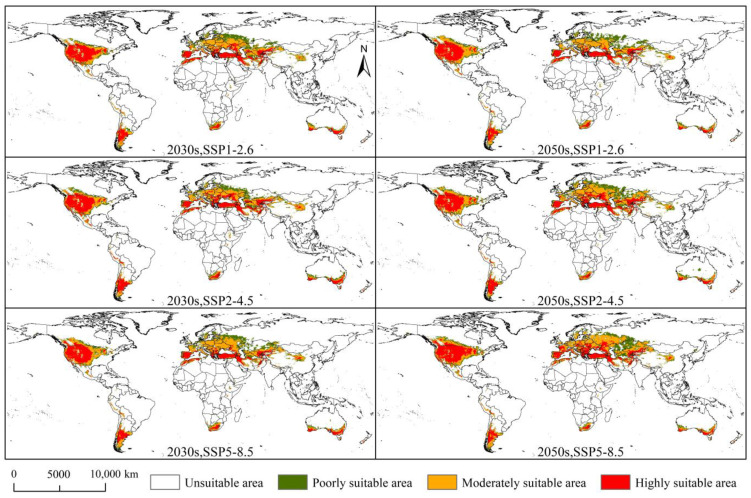
Potential suitable areas for *Diuraphis noxia* under future climatic scenarios.

Compared with the current climatic scenario, the total suitable area increased in the 2030s and 2050s under the SSP1-2.6, SSP2-4.5, and SSP5-8.5 climatic scenarios (Figure 7). The highly, moderately, and poorly suitable areas increased in addition to the highly suitable area under SSP5-8.5, in the 2030s, and SSP1-2.6 under the 2050s climatic scenarios. Under future climatic scenarios, *D. noxia* was mainly located in central and western Asia, western Europe, and central and southwestern North America. 

In the 2030s, the total, highly, moderately, and poorly suitable areas of *D. noxia* increased under the SSP1-2.6 and SSP2-4.5 climatic scenarios (Table S2). Under the SSP5-8.5 climatic scenario, the total, moderately, and poorly suitable areas of *D. noxia* reached the maximum values of 2619.24 × 10^4^ km^2^, 1075.61 × 10^4^ km^2^, and 710.87 × 10^4^ km^2^, respectively, and the highly suitable area reached a minimum value of 832.76 × 10^4^ km^2^. Further, under the SSP2-4.5 climatic scenario, the highly suitable area reached a maximum value of 986.29 × 10^4^ km^2^. In the 2050s, the total, highly, moderately, and poorly suitable areas of *D. noxia* increased under the SSP2-4.5 and SSP5-8.5 climatic scenarios (Table S2). Under the SSP5-8.5 climatic scenario, the total, highly, and moderately suitable areas of *D. noxia* reached maximum values of 2950.40 × 10^4^ km^2^, 1042.84 × 10^4^ km^2^, and 1195.36 × 10^4^ km^2^, respectively, and the poorly suitable area reached a minimum value of 712.21 × 10^4^ km^2^. In the SSP2-4.5 climatic scenario, the poorly suitable area reached a maximum value of 754.01 × 10^4^ km^2^.

### 3.5. Changes in the Potential Suitable Areas under Different Climatic Scenarios

**Figure 8:**
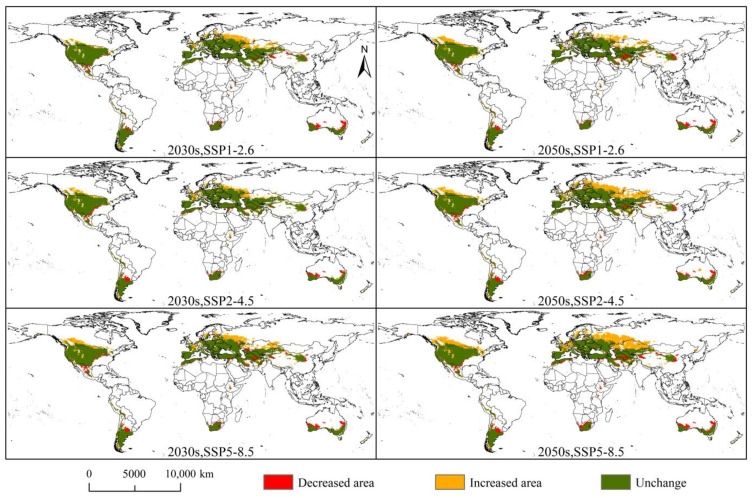
Changes in the *Diuraphis noxia* potential suitable areas under different climatic scenarios.

The changes in the potential suitable areas of *D. noxia* in the 2030s and 2050s under the SSP1-2.6, SSP2-4.5, and SSP5-8.5 climatic scenarios are shown in Figure 8. Under the SSP1-2.6, SSP2-4.5, and SSP5-8.5 climatic scenarios, the areas of *D. noxia* increased by 432.87 × 10^4^ km^2^, 345.92 × 10^4^ km^2^, and 516.22 × 10^4^ km^2^ in the 2030s, respectively (Table 2), and were mainly distributed in central Asia (Kazakhstan), western Europe (Western Russia, Finland, and France), and North America (Canada), whereas the areas that decreased by 189.37 × 10^4^ km^2^, 192.63 × 10^4^ km^2^, and 251.77 × 10^4^ km^2^, respectively (Table 2), were mainly distributed in southern South America (Argentina), southern Africa (South Africa), and southeastern and southwestern Oceania (Australia). The potential suitable areas of *D. noxia* increased by 445.19 × 10^4^ km^2^, 694.56 × 10^4^ km^2^, and 882.91 × 10^4^ km^2^ under the SSP1-2.6, SSP2-4.5, and SSP5-8.5 climatic scenarios in the 2050s, respectively (Table 2), and were mainly distributed in central Asia (Kazakhstan), western Europe (Russia, Finland, France, and Sweden), and central North America (Canada). Moreover, the potential suitable areas of *D. noxia* decreased by 246.48 × 10^4^ km^2^, 225.94 × 10^4^ km^2^, and 286.38 × 10^4^ km^2^ under the SSP1-2.6, SSP2-4.5, and SSP5-8.5 climatic scenarios, respectively (Table 2), and were mainly distributed in southern South America (Argentina), southern Africa (South Africa and Morocco), and southeastern and southwestern Oceania (Australia). 

### 3.6. Migration of Centroid in the Potential Suitable Areas of Diuraphis noxia under Climate Change

**Figure 9:**
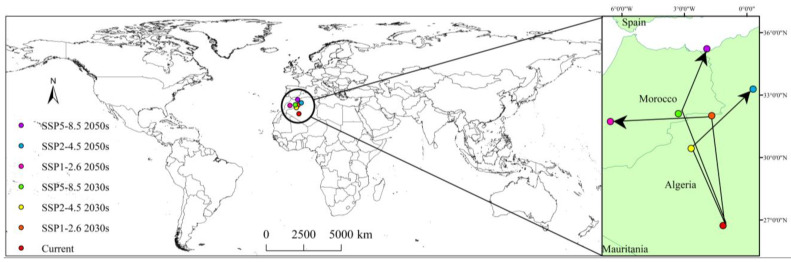
Centroid migration of potential suitable areas of *Diuraphis noxia* under current and future climatic scenarios.

Under the current and future climatic scenarios, the centroid of potential suitable areas of *D. noxia* showed a tendency to migrate northward (Figure 9). The current centroid of potential suitable areas was located in Algeria (1°8′42.235″ W, 26°43′34.733″ N). In the 2030s and 2050s climatic scenarios, under SSP1-2.6, the centroid migrated to Algeria (1°41′8.558″ W, 32°0′18.007″ N) and Morocco (6°32′52.639″ W, 31°43′29.344″ N), respectively; under SSP2-4.5, to Algeria (2°40′16.215″ W, 30°25′48.352″ N) and Algeria (0°17′22.889″ E, 33°16′54.021″ N), respectively; and under SSP5-8.5, to Morocco (3°17′22.889″ W, 32°6′6.814″ N) and Mediterranean region (1°55′19.329″ W, 35°13′2.015″ N), respectively. The centroid of the potential suitable areas of *D. noxia* migrated 589.27, 437.93, and 632.72 km from the current climatic scenario under SSP1-2.6, SSP2-4.5, and SSP5-8.5, respectively, in the 2030s climatic scenarios, and 763.79, 741.84, and 947.00 km from the current climatic scenario to SSP1-2.6, SSP2-4.5, and SSP 5-8.5, respectively, in the 2050s climatic scenarios. Thus, the centroid of the potential suitable area showed a trend toward higher latitudes. 

## 4. Discussion

Invasive crop pests pose a significant threat to agricultural production and food security [47]. *Diuraphis noxia* is one of the most devastating ICPs [48] and significantly damages barley and wheat worldwide [28,49]. Predicting the potential global geographical distribution of *D. noxia* is crucial for early warning and management. In this study, an optimized MaxEnt model was used to predict the potential global geographical distribution and its changes under current and future climatic scenarios. In addition to providing a basis for controlling *D. noxia*, the study also assists indirectly in maintaining food security. 

Insects are ectothermic animals that are highly sensitive to temperature changes [50]. Temperature can directly affect their growth, development, and survival [51,52], and when indirectly combined with other factors, it can influence their reproduction [53]. For example, *Nezara viridula* has a much higher overwintering survival with increasing temperatures [54]. Our results indicated that temperature was a significant factor affecting the potential global geographical distribution of *D. noxia.* The survival of *D. noxia* increased and then decreased with increasing annual mean temperature (Bio1) and temperature annual range (Bio7). A temperature threshold exists in the growth and development of *D. noxia*, before which survival of *D. noxia* increases with rising temperature (for example, the survival of *D. noxia* is higher at 18–21 °C than at 5–15 °C [55]) and above which the survival decreases with rising temperature [56]. Further, when the temperature drops to below 0 °C, the *D. noxia* population catastrophically declines, and when the temperature drops to −15 °C, *D. noxia* mortality occurs in a short time [57,58,59,60]. For instance, in northern USA and Canada, the overwintering survival of *D. noxia* can drop to zero if the temperature is sufficiently low and the low temperature is maintained for relatively long periods [61,62]. Based on previous studies, our results were verified, demonstrating that our results were reasonable and accurate. 

Climate change is a significant limiting factor affecting the potential global geographical distribution of ICPs, thereby increasing the risk of invasion and expansion of their infestation range [63,64,65]. For example, *Spodoptera littoralis*, *Naupactus leucoloma*, and *Bemisia tabaci* are more widespread due to climate change [66,67,68]. Our results indicated that the potential suitable areas of *D. noxia* will increase with climate change in the future and that the increased areas will vary regionally. The major wheat-producing regions in the world, such as Russia, Canada, the USA, and France, are the main regions where the area of *D. noxia* is predicted to increase under the future climatic scenarios. This would seriously threaten the wheat industry in these countries along with global food security. Therefore, these countries should establish detection, monitoring, and control measures to prevent invasion of *D. noxia*. Moreover, potential suitable areas of *D. noxia* migrated to higher latitudes. The population density of *D. noxia* can increase with increasing CO_2_ concentration [69], and thus, with global climate warming, the population of *D. noxia* can increase dramatically. Therefore, in response to climate change, *D. noxia*, on the one hand, searches for new areas for survival; on the other hand, to solve the problems of interspecific competition due to excessive population, less food, and narrow habitat areas, *D. noxia* will migrate to higher latitudes.

Our results also indicated that central South America, central Africa, eastern Asia, and central Oceania are unsuitable for *D. noxia*, indicating that temperature is not the only factor limiting the potential global geographical distribution of *D. noxia*, and host plant abundance, wheat species, and other factors also play a role. Generally, when wheat or other major host plants are harvested, *D. noxia* shifts to weeds; in spring wheat-growing areas, large *D. noxia* populations exist and abundant hosts are present, whereas in winter wheat-growing areas, *D. noxia* populations are small and have difficulty overwintering [70]. 

*Diuraphis noxia* can disperse not only over short distances by migrations, but also over long distances through international trade of crops, such as wheat, barley, rye, and oats [71,72,73,74]. Therefore, in the future climatic scenario, these countries with increased potential suitable areas, particularly, the UK, Russia, Canada, Sweden, and France, which showed a relatively large area of increase, should be alert to the import of the host plants and strengthen the warning and management system to prevent secondary invasion. Moreover, *D. noxia* can suck sap from the phloem of infested crops, thereby causing yield losses [30,33]. Major barley and wheat cultivation areas worldwide, including in Canada, Australia, and Russia, were suitable for *D. noxia*, thus presenting a potential threat to the global grain industry. *Diuraphis noxia* is widely distributed in central North America, western Asia, and most of Europe. Based on the above invasion risk, these countries should not only strengthen the management of *D. noxia,* but also implement effective control measures, including cultural, chemical, and biological measures, to eradicate *D. noxia* [22]. Cultural measures include postponing the sowing of host plants, intercropping, and crop substitution [75]. Chemical measures mainly involve the use of insecticides, such as imidacloprid and chlorpyrifos [75]. Biological measures involve the use of natural enemies of *D. noxia*, including predatory insects and parasitic wasps [75]. Although these control measures can control *D. noxia* growth, they all have drawbacks, such as the slow effect of the cultural measures, the potential of chemical measures to damage the crop itself, and the long research period of biological measures before their implementation. Therefore, different control measures should be used rationally to control *D. noxia*.

## 5. Conclusions

In this study, we used an optimized MaxEnt model based on 533 global occurrence records and 9 bioclimatic variables to predict the potential global geographical distribution of *D. noxia*. Evaluation of the model accuracy showed that the optimized MaxEnt model had high accuracy. Under the current climatic scenario, *D. noxia* was mainly distributed in North America, Asia, and Europe. The potential suitable areas of *D. noxia* increased under the future climatic scenarios in the context of climate change, and *D. noxia* showed a tendency to migrate to higher latitudes. Moreover, temperature was a significant factor influencing the potential global geographical distribution of *D. noxia*. Therefore, wheat- and barley-producing countries, such as France, Sweden, Canada, Australia, and Russia, which are suitable for the survival of *D. noxia*, should strengthen quarantine measures to prevent secondary invasion while implementing control measures to eradicate *D. noxia*. Overall, this study provides a theoretical basis for the management and control of *D. noxia*.

## Figures and Tables

**Table 1:** Bioclimatic variables used for model construction.

Index	Description	Unit
Bio1	Annual Mean Temperature	°C
Bio2	Mean Diurnal Temperature Range	°C
Bio7	Temperature Annual Range	°C
Bio8	Mean Temperature of Wettest Quarter	°C
Bio12	Annual Precipitation	mm
Bio15	Precipitation Seasonality	mm
Bio17	Precipitation of Driest Quarter	mm
Bio18	Precipitation of Warmest Quarter	mm
Bio19	Precipitation of Coldest Quarter	mm

**Table 2:** Changes in potential suitable areas of *Diuraphis noxia* under future climatic scenarios (10^4^ km^2^).

Period	Decreased	Increased	Unchanged
2030s, SSP1-2.6	189.37	432.87	2168.47
2030s, SSP2-4.5	192.63	345.92	2165.64
2030s, SSP5-8.5	251.77	516.22	2105.98
2050s, SSP1-2.6	246.48	445.19	2111.17
2050s, SSP2-4.5	225.94	694.56	2132.05
2050s, SSP5-8.5	286.38	882.91	2072.20

## Data Availability

The data presented in this study are available in this article.

## Supplementary Materials

The following supporting information can be downloaded at: https://www.mdpi.com/article/10.3390/insects14050425/s1, Table S1: Bioclimatic variables correlated with the distribution of *Diuraphis noxia*; Table S2: Potential suitable areas for *Diuraphis noxia* under different climatic scenarios (10^4^ km^2^); Figure S1: Three emission scenarios; Figure S2: Contribution rate of bioclimatic variable.
