# Supplementary material for: Estimating the Global Geographical Distribution Patterns of the Invasive Crop Pest Diuraphis noxia Kurdjumov under Current and Future Climatic Scenarios
Source: Insects. 2023 Apr 29;14(5):425. doi: 10.3390/insects14050425 (PMC10231117; doi:10.3390/insects14050425)
Supplement: Supplementary file 1 [file insects-14-00425-s001.zip › insects-2338126-supplementary.pdf]

Supplementary material

Table S1. Bioclimatic variables correlated with the distribution of *Diuraphis noxia*

Variable	Description	Unit
Bio1	Annual mean temperature	℃
Bio2	Mean diurnal temperature area	℃
Bio3	Isothermality (bio2/bio7) (*100)	-
Bio4	Temperature seasonality (standard deviation*100)	℃
Bio5	Max temperature of warmest month	℃
Bio6	Min temperature of coldest month	℃
Bio7	Temperature annual range	℃
Bio8	Mean temperature of wettest quarter	℃
Bio9	Mean temperature of driest quarter	℃
Bio10	Mean temperature of warmest quarter	℃
Bio11	Mean temperature of coldest quarter	℃
Bio12	Annual precipitation	mm
Bio13	Precipitation of wettest month	mm
Bio14	Precipitation of driest month	mm
Bio15	Precipitation seasonality (coefficient of variation)	-
Bio16	Precipitation of wettest quarter	mm
Bio17	Precipitation of driest quarter	mm
Bio18	Precipitation of warmest quarter	mm
Bio19	Precipitation of coldest quarter	mm

Table S2. Potential suitable areas for *Diuraphis noxia* under different climatic scenarios (10^4 km^2).

Period	Highly	Moderately	Poorly	Total suitable
Current	899.03	873.31	580.70	2353.05
2030s, SSP1-2.6	911.34	1000.65	686.51	2598.50
2030s, SSP2-4.5	986.29	922.68	599.87	2508.85
2030s, SSP5-8.5	832.76	1075.61	710.87	2619.24
2050s, SSP1-2.6	851.28	979.19	723.06	2553.53
2050s, SSP2-4.5	987.19	1082.73	754.01	2823.93
2050s, SSP5-8.5	1042.84	1195.36	712.21	2950.40

Emission	Description
SSP1-2.6	SSP1(Low forcing scenario) Upgrade to RCP2.6 scenario based on (Radiative forcing reaches 2.6 W/m^2 in 2100)
SSP2-4.5	SSP2(Intermediate forcing scenario) Upgrade to RCP4.5 scenario based on (Radiative forcing reaches 4.5 W/m^2 in 2100)
SSP5-8.5	SSP5(High forcing scenario) Upgrade to RCP8.5 scenario based on (Radiative forcing reaches 8.5 W/m^2 in 2100)

Figure S1. Three emission scenarios.

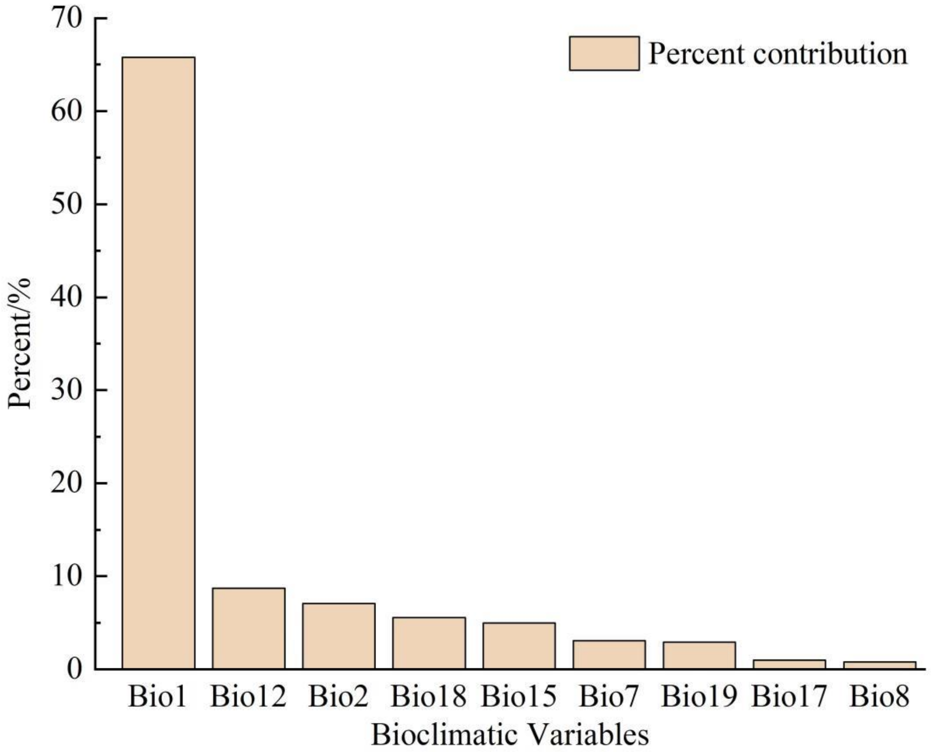

Figure S2. Contribution rate of bioclimatic variables.
